# Net-spinning caddisflies create denitrifier-enriched niches in the stream microbiome

**DOI:** 10.1038/s43705-023-00315-8

**Published:** 2023-10-17

**Authors:** Anthony D. Bertagnolli, Andrew J. Maritan, Benjamin B. Tumolo, Samuel F. Fritz, Hayley C. Oakland, Elizabeth J. Mohr, Geoffrey C. Poole, Lindsey K. Albertson, Frank J. Stewart

**Affiliations:** 1 Department of Microbiology & Cell Biology, Montana State University, Bozeman, MT 59717 USA; 2 Department of Ecology, Montana State University, Bozeman, MT 59717 USA; 3 Department of Land Resources and Environmental Sciences, Montana State University, Bozeman, MT 59717 USA; 4 Montana Institute on Ecosystems, Montana State University, Bozeman, MT 59717 USA; 5 Center for Microbial Dynamics and Infection, School of Biological Sciences, Georgia Institute of Technology, Atlanta, GA 30332 USA

**Keywords:** Microbial ecology, Biogeochemistry

## Abstract

Larval net-spinning caddisflies (Hydropsychidae) function as ecosystem engineers in streams where they construct protective retreats composed of organic and inorganic material affixed with silk filtration nets that alter streambed hydrology. We hypothesized that hydropsychid bio-structures (retreats, nets) are microhabitats for microbes with oxygen-sensitive metabolisms, and therefore increase the metabolic heterogeneity of streambed microbial assemblages. Metagenomic and 16 S rRNA gene amplicon analysis of samples from a montane stream (Cherry Creek, Montana, USA) revealed that microbiomes of caddisfly bio-structures are taxonomically and functionally distinct from those of the immediately adjacent rock biofilm (~2 cm distant) and enriched in microbial taxa with established roles in denitrification, nitrification, and methane production. Genes for denitrification, high oxygen affinity terminal oxidases, hydrogenases, oxidative dissimilatory sulfite reductases, and complete ammonia oxidation are significantly enriched in caddisfly bio-structures. The results suggest a novel ecosystem engineering effect of caddisflies through the creation of low-oxygen, denitrifier-enriched niches in the stream microbiome. Facilitation of metabolic diversity in streambeds may be a largely unrecognized mechanism by which caddisflies alter whole-stream biogeochemistry.

Aquatic insects can physically engineer stream ecosystems by influencing bed hydraulics and sediment transport [1–3]. Net-spinning caddisflies (family Hydropsychidae) are abundant in streams, reaching densities in the 1000 s per cubic meter [4]. Hydropsychid larvae spin silk nets for filter feeding and construct protective retreats from silk, sand, and vegetative material (Fig. 1A). Larval retreats and nets (hereafter, “bio-structures”) occupy the interstitial spaces among streambed sediment grains, increasing resistance to water flow through the gravel and promoting colonization of other invertebrates [5–7]. These modifications may affect biochemical flux in streams, as up to 90% of stream nutrient cycling occurs in streambed gravels [8]. However, the potential for caddisfly ‘ecosystem engineering’ to influence stream microbiomes and associated biochemical fluxes remains understudied. Aquatic biofilms maintain steep oxygen gradients that promote dissimilatory nitrogen processes [9–11]. Given the damping effect of bio-structures on water flow in the hyporheic zone and their established role in creating oxygen gradients, we hypothesized that bio-structures create novel niches for biofilms enriched in anaerobic metabolisms of biochemical consequence to the stream ecosystem, specifically the process of bacterial denitrification, the predominant nitrogen removal pathway in freshwater lotic environments [12].

**Fig. 1:**
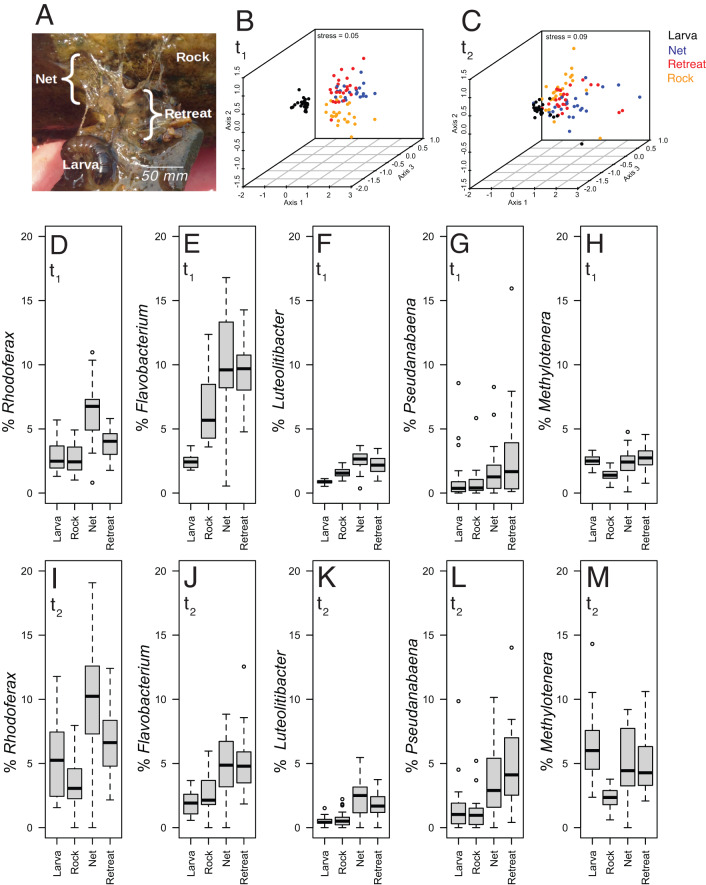
Microbiome sampling schematic, multivariate analyses, and relative abundances of dominant taxa. Microbiome samples were collected from net-spinning caddisfly (Hydropsychidae) larvae and their filtration nets and retreats (“bio-structures”) and compared to those from adjacent rock-attached (control) biofilms (**A**). Microbiome taxonomic composition was compared by nonmetric multidimensional scaling (NMDS) analysis of unweighted UniFrac distances based on t_1_ (April 7, **B**) and t_2_ (June 2, **C**) 16 S rRNA gene ASV datasets. Final stress values for 3-d solutions are listed in (**B**, **C**). Mean percentage abundances of five dominant genera (1% or greater average 16 S rRNA gene amplicon relative abundance across t_1_ and t_2_) (**D**–**M**) with significant variation (*p* < 0.05; ANOVA with Tukey’s HSD post-hoc testing) in abundance between either nets or retreats versus rock biofilms at t_1_ (**D**–**H**) and t_2_ [42]. Genera are *Rhodoferax* (**D**, **I**), *Flavobacterium* (**E**, **J**), *Luteolibacter* (**F**, **K**), *Pseudanabaena* (**G**, **L**), and *Methylotenera* (**H**, **M**). Vertical axes are percentage abundance with identical scales. In (**D**–**M**), the bold line is the sample mean; the boxed region is the interquartile range (IQR); top and bottom whiskers indicate [Q3 + 1.5*IQR] and [Q1–1.5*IQR] respectively; and outliers are marked by open circles.

We therefore investigated the taxonomic composition of larval caddisfly-associated prokaryotic microbiomes in a 3rd order montane stream (Cherry Creek, Montana, USA) at a single reach (i.e., one site, ~20 m length) on two dates during spring snowmelt: April 7th (t_1_) and June 2nd (t_2_), 2021. We sampled during spring in order to observe larvae during their presumed maximal activities, which typically occur during mid-spring prior to peak flow rates; exact dates were chosen to avoid dangerous flow rates. Using analysis of 16 S rRNA gene amplicon sequence variants (ASVs), we compared microbiomes associated with larvae (homogenized whole larva, *n* = 24 and 27 for t_1_ and t_2_, respectively), silk nets (*n* = 23, 24), retreats (*n* = 24, 26), and non-caddisfly-associated rock biofilms (~2 cm distant from each caddisfly retreat; *n* = 24, 25). We also contextualized these microbiomes against a limited number of stream water (*n* = 1 and 3 at t_1_ and t_2_) and surficial sediment microbiomes (*n* = 4 at both t_1_ and t_2_). Analysis of similarity (ANOSIM), permutational multivariate analysis of variance (PERMANOVA), and non-metric multidimensional scaling (NMDS) based on unweighted UniFrac distances (chloroplast and mitochondrion ASVs removed; samples with fewer than 500 sequences removed; median sequence depth: 30,856; range 528 to 113,697) revealed that microbiome composition varied significantly according to sample type at both t_1_ (R = 0.55, *p* < 0.01, ANOSIM) and t_2_ (R = 0.33, *p* < 0.01, ANOSIM) (Fig. 1B, C, Tables S1, S2); analysis using weighted UniFrac and Bray Curtis dissimilarity metrics yielded consistent results (Tables S1, S2). These analyses also detected significant variation when bio-structure microbiomes (nets/retreats, evaluated independently and as combined datasets) were compared directly to those of rock control microbiomes (i.e., larval microbiomes excluded, *p* < 0.01 for all metrics, ANOSIM, Table S1, Fig. S1), with highest R values (0.35) for unweighted UniFrac-based comparisons between t_2_ net versus rock microbiomes. Supervised learning through Random Forest analysis supported the ANOSIM/PERMANOVA observations, accurately predicting differences in community structure among larval, biostructure (nets/retreats) and rock control microbiomes. Out-of-bag error rates (OOB, a reflection of sample type classification accuracy) were 14.7 and 21.6% for t_1_ and t_2_ independently, with class errors of 0.00 (t_1_) and 0.04 (t_2_) for classification of rock controls versus all other sample types, indicating the model could accurately discriminate caddisfly-influenced (larva, nets, retreats) from non-influenced rock microbiomes with close to 100% accuracy (Table S3). When larval microbiome datasets were removed, OOB and class errors for differentiating rock control from nets/retreats were 19.7% and 0.00, respectively, at both t_1_ and t_2_. Across all sample types, t_1_ microbiomes differed significantly from t_2_ microbiomes (R = 0.30–0.44, *p* < 0.01, ANOSIM). However, sample type-specific community signatures were less visually evident at t_2_ compared to t_1_ (Fig. 1B vs. C), consistent with an increase in inter-sample variation (dispersion) across all sample types from t_1_ to t_2_ (Figs. S2, S3). Taken together, these results confirm that caddisfly larvae and caddisfly-associated bio-structures harbor microbiomes distinct from those of adjacent rock, despite these microbiomes being dynamic over time.

We identified 81 and 20 prokaryotic genera enriched in bio-structures (in either nets or retreats, or in both) compared to rock biofilms at t_1_ and t_2_, respectively (*p* < 0.05; ANOVA with Tukey’s HSD post hoc test). Of these genera, only 5 displayed mean percentage abundances greater than 1% in any of the sample types and were significantly enriched in bio-structures at both timepoints. These five included *Flavobacterium* (class Bacteroidia), *Pseudanabaena* (class Oxyphotobacteria), *Luteolibacter* (class Verrucomicrobiae), *Rhodoferax* (class Betaproteobacteria), and *Methylotenera* (class Betaproteobacteria) (Fig. 1D–M). The mean decrease in Gini coefficient (MDG, generated from Random Forest decision trees) was 0.62, 1.41, 0.99, 2.39, and 0.911 for *Flavobacterium*, *Pseudanabaena*, *Luteolibacter*, *Rhodoferax*, and *Methylotenera*, respectively, compared to a median MDG of 0.012 across all taxa, thereby providing further support for these genera in driving differences between bio-structure and rock control microbiomes (Table S4). *Flavobacterium* and *Luteolibacter* spp. are common stream representatives with diverse chemoheterotrophic roles [13], including the degradation of complex polymers [14], whereas *Pseudanabaena* spp. are filamentous phototrophs generally associated with bloom formation [15]. *Rhodoferax* spp. are metabolically diverse, with representatives capable of anoxygenic photoheterotrophy and iron reduction, as well as aerobic respiration. Members of *Methylotenera* from lake sediments have been reported to anaerobically couple methylotrophy to nitrate reduction [16].

Nets and retreats were also significantly enriched in several other taxa of potential relevance to chemical cycling, notably aerobic ammonia-oxidizing Nitrososphaerota (*Candidatus* Nitrosopumilus) and methanogens (genera *Methanosarcina, Methanobacterium*, and *Methanoregula*), albeit at lower levels (less than 1% mean abundance across sample types) (Fig. S5). The aerobic, nitrifying bacterial genus *Nitrospira* was also enriched in retreats (~0.2%) compared to rocks, although not significantly. The ecology of aerobic nitrifiers is relatively understudied in streams compared to other aquatic systems. *Nitrospira* spp. capable of both nitrite oxidation and complete ammonia oxidation to nitrate (comammox) have been described in a large river system [17], but the diversity of the group is relatively unknown for many lotic systems. Methanogen taxonomic composition in streams is seemingly influenced by stream order, with *Methanosarcina* more common in warm, oxygen-poor streams and *Methanobacterium* more common in colder, oxygen-rich waters [18]. It remains uncertain how these taxa vary in abundance and activity at the microhabitat level.

These taxonomic trends prompted us to test for the relative abundance of microbial genes representing biogeochemically relevant metabolisms. Metagenomes from each of the four sample types at t_1_ (4 datasets per sample type, 16 total, see Table S5 for sequencing and assembly statistics) were queried against a database of 50 marker genes of trace gas (including methane) metabolism, dissimilatory sulfur and nitrogen metabolisms, carbon fixation, photosynthesis, and aerobic respiration (SI Materials and Methods). This database was compiled and vetted in prior studies of microbial biogeochemical diversity, with genes selected based on their use to detect ecologically relevant metabolisms [19–21] (see SI for details). Our analysis revealed a subset of genes enriched in caddisfly-associated microhabitats. Notably, genes mediating each of the four steps of denitrification—encoding nitrate (*narG*), nitrite (*nirS* and *nirK*), nitric oxide (*norB*) and nitrous oxide (*nosZ*) reductases—were consistently enriched in nets and retreats compared to rocks (Fig. 2A-E), with *narG* and *norB* significantly enriched (*p* < 0.05; ANOVA with Tukey’s HSD post-hoc test) (Fig. 2, Fig. S6). This pattern was observed regardless of whether assembled (contig) or non-assembled sequences were used as queries. Some of these denitrification genes could be assigned to specific metagenome-assembled genomes (MAGs) affiliated with the genera *Rhodoferax* (*narG* and *norB*), *Flavobacteriaceae* (*nos*Z), *Spirosomaceae* (*nosZ*), and the family Rhizobiaceae (*nirK*). These four MAGs consistently peaked in representation in the metagenomes from nets and retreats (Fig. S7; see SI for abundance calculations). None of these MAGs were complete (27-92% completeness, Table S6), making it challenging to predict their potential for complete denitrification. Indeed, in all samples, the community *nosZ* gene pool was dominated by ‘atypical’ clade II sequence variants (Fig. S8), which are most often recovered from genomes that lack a complete set of genes for canonical denitrification and are associated with higher affinity for N_2_O compared to ‘typical’ *nosZ* variants [22, 23]. Nets and retreats were also significantly enriched in genes for *cbb*_3_ terminal oxidases (*p* < 0.05) (Fig. 2F), which were also detected in both *nosZ*-containing MAGs. *cbb*_3_ oxidases are known to be induced under low oxygen and play an important role regulating denitrification in certain taxa, including known biofilm formers (e.g., *Pseudomonas aeruginosa*) [24, 25]. Taken together, these functional gene and MAG taxonomic trends suggest that caddisfly activity may enrich for denitrifying taxa associated with low oxygen availability.

**Fig. 2:**
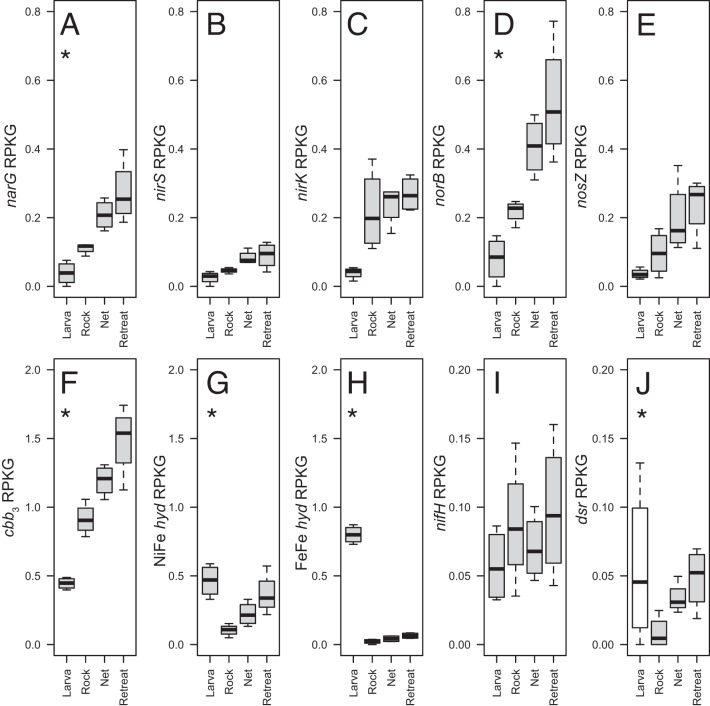
Mean proportional abundance, expressed as reads per kilobase genome equivalent (RPKG), of genes enriched in either retreats or nets versus rock biofilms. Genes displaying significant enrichment in either retreats or nets versus rock biofilms (*p* < 0.05; ANOVA with Tukey’s HSD post-hoc testing) are indicated with asterisks (*). Panels (**A**–**E**) show genes encoding enzymes of the denitrification pathway: nitrate reductase (*narG*) (**A**), cytochrome *cd*-1 nitrite reductase (*nirS*) (**B**), copper containing nitrite reductase (*nir*K) (**C**), nitric oxide reductase (*nor*B) (**D**), nitrous oxide reductase (*nosZ*) (**E**). Y-axes are identical in scale in (**A**–**E**). Panels (**F**–**J**) show genes encoding high affinity cytochrome-c oxidase (*cbb*_3_) (**F**), nickel-iron hydrogenase (NiFe-*hyd*) (**G**), iron-iron hydrogenase (FeFe-*hyd*) (**H**), nitrogenase (*nifH*) (**I**), and reductive (white) and oxidative [43] dissimilatory sulfite reductases (*dsr*) (**J**). For panels (**F**–**J**), the vertical axes have the same units but different ranges. In (**A**–**J**), the bold line is the sample mean; the boxed region is the interquartile range (IQR); top and bottom whiskers indicate [Q3 + 1.5*IQR] and [Q1–1.5*IQR] respectively; and outliers are marked by open circles.

Other biochemically relevant marker genes also varied in representation across sample types. Genes encoding NiFe- and FeFe-hydrogenases were at peak abundance in larval microbiomes, likely due to the presence of intestinal bacteria conducting fermentation or H_2_-oxidation. Interestingly, these genes were also significantly enriched in nets and retreats compared to rock biofilms (Fig. 2G, H, respectively), as were genes encoding oxidative dissimilatory sulfite reductases, with the latter suggesting a role for inorganic sulfur cycling in bio-structure biofilms (Fig. 2J and SI Text). While genes linked to dissimilatory nitrogen metabolism were well represented in our data, marker genes for nitrogen fixation (*nifH*) were rare and did not vary substantially in frequency among sample types (Fig. 2I).

Certain genes of nitrification were also enriched in biostructures. Notably, ammonia monooxygeanse (*amoA*) genes were recovered at peak abundance in retreats (Fig. S9, S10), with these *amoA* genes associated phylogenetically with comammox *Nitrospira* (Fig. S9). No Thaumarchaeal or betaproteobacterial *amoA* fragments were recovered. Genes matching *Nitrospira* alpha and beta nitrite oxidoreductases (*nxrA*, *nxrB*), hydroxylamine oxidoreductase (*haoB*), and ATP-citrate lyase (*aclB*) were observed at peak abundance in retreats (Fig. S10). Two of these genes (*nxrA, haoB*) were also identified on a retreat-associated MAG (27% complete) that was phylogenetically associated with *Nitrospira* Clade B (SI Table 6) and closely related to MAGs from freshwater sand filters (Fig. S11) [26, 27]. This MAG also contained a 16 S rRNA gene with 98% similarity to the dominant *Nitrospira* ASV in the amplicon data (Fig. S9). *Nitrospira* bacteria are often observed in biofilms [28, 29], with their biofilm association potentially related to oxygen [30]. Indeed, both comammox and nitrite-oxidizing *Nitrospira* use an oxygen-sensitive carbon fixation pathway (reverse TCA cycle) that differs from Thaumarchaeal (hydroxypropionate/hydroxybutyrate cycle) and betaproteobacterial (Calvin Benson cycle) nitrifying counterparts [31, 32]. Together, our metagenome and amplicon data indicate an enrichment of *Nitrospira* in bio-structures, suggesting a contribution of comammox to nitrification and a role for ecosystem-engineering caddisflies in structuring nitrifier diversity in lotic systems.

Ecosystem engineers such as net-spinning caddisflies exert their engineering effects primarily through physical habitat modifications that positively affect surrounding taxa [4]. The beneficiaries of ecosystem engineers span taxonomic and trophic levels, including microbes [1]. While microbes are among the taxa with the strongest response to engineering activities, they are rarely investigated in this context – a recent meta-analysis examined 340 studies of positive interactions in freshwater environments, finding that only 2.4% of studies considered bacteria or archaea as beneficiaries of interaction [33]. A small number of studies have evaluated the internal microbiomes of ecosystem engineering aquatic insects—for example, testing the role of feeding guild (i.e., shredder, decomposer, predator, collector/gatherer) and host identity in shaping microbiome diversity [34–38]. However, the extent to which aquatic insects shape the microbiology of the surrounding environment remains relatively unknown. The data presented here indicate that biofilms of caddisfly bio-structures are taxonomically and functionally distinct from those of adjacent rock surfaces. Notably, an enrichment of denitrification and comammox genes in bio-structures suggests a direct linkage between ecosystem engineers and microbially-mediated nitrogen cycling, while enrichment of methanogens suggests linkages to greenhouse gas flux. The magnitude of caddisfly-associated chemical flux is unknown but may be substantial in montane streams where caddisfly abundance is high [39]. Measuring this contribution is critical given the increasing potential for temperature, drought, nutrient, and other environmental stress to stream ecosystems and their resident engineers [40, 41].

### Supplementary information

Supplementary Information Document

Supplementary Information Tables

## Data Availability

All sequence data generated in this study are available through the NCBI Sequence Read Archive under BioProject PRJNA834817.
