# Supplementary material for: Net-spinning caddisflies create denitrifier-enriched niches in the stream microbiome
Source: ISME Commun. 2023 Oct 17;3:111. doi: 10.1038/s43705-023-00315-8 (PMC10582121; doi:10.1038/s43705-023-00315-8)
Supplement: Supplementary file 1 — Supplementary Information Document [file 43705_2023_315_MOESM1_ESM.docx]

SUPPLEMENTARY INFORMATION

***Net-spinning caddisflies create denitrifier-enriched niches in the stream microbiome***

Anthony D. Bertagnolli, Andrew J. Maritan, Benjamin B. Tumolo, Samuel F. Fritz, Hayley C. Oakland, Elizabeth J. Mohr, Geoffrey C. Poole, Lindsey K. Albertson, Frank J. Stewart

This document contains:

Methods (pages 1-7)

Supplementary Discussion (page 8)

Supplementary figure (11) and table (6) legends (pages 9-11)

Supplementary figures and tables** (pages 12-26)

References (pages 27-29)

***Tables S4 and S6 are too large to be included here and are uploaded as separate Excel files*

**METHODS**

**Site description and sample collection**

Caddisfly-associated samples were collected on 7 April 2021 (t_1_) and 2 June 2021 (t_2_) from Cherry Creek, Red Bluff, Montana (45.612309, -111.516983). Cherry Creek is a third order stream with a streambed comprised of coarse sand, pebbles, gravel, and cobble. The Cherry Creek watershed is owned by Turner Enterprises, Inc, and managed for conservation. The immediate riparian zone consists predominantly of grasses, low woody plants, and sparse trees. This sampling site (or river) was selected because it is not in close proximity to agricultural or urban influences.

Samples were collected between 1050-1630 hours (Mountain Standard Time) from the same approximately 20-m long reach on both collection dates. The average water depth was 0.15 and 0.21 m in April and June, respectively. The stream was wadable on both dates. Water temperatures were estimated at 6-8°C in April (inferred from measurements nearby in the Gallatin and Maddison rivers) and 10-12 °C in June (measured at the sampling site). Flow and suspended particle load were higher in June than April but were not quantified.

On both dates, samples from larval Hydropsyche spp. (hereafter “caddisfly”)-associated microhabitats and controls were collected using methods similar to those used previously [[1](#_ENREF_1)]. Briefly, a cobble (~20-40 cm) was selected haphazardly from the river from beneath visually identifiable riffles. When an inhabited caddisfly-retreat-net complex was identified, we collected four samples per cobble: 1) the larval caddisfly (larva), 2) its fixed rock shelter (retreat) 3) its silk foraging net [[2](#_ENREF_2)], 4) the cobble biofilm away from the fly-retreat-net complex (rock control). Larvae, retreats, and nets were collected with sterile forceps and placed into separate 2 mL cryovials (Corning Inc., Corning, NY) each containing RNA/DNA stabilizing buffer (25 mM sodium citrate, 10 mM EDTA and 70 g ammonium sulfate per 100 ml solution, pH 5.2). Rock biofilms were collected by firmly scrubbing a sterile cotton swab over a small (~2 cm^2^) area approximately 3-4 cm from the net-retreat complex for 20 seconds; brown discoloration of the swab head was evident after each scrubbing. The swab head was then cut off and placed in a cryovial with RNA/DNA stabilizing buffer. This procedure was repeated with different cobbles for each set of four samples, yielding 96 samples in April (24 larvae, 25 retreats, 23 nets, 24 rock swabs) and 105 in June (27 larvae, 26 retreats, 25 nets, 27 rock swabs). Numbers reflect those samples that yielded sufficient DNA yield (see *DNA extraction*) and generated 16S rRNA gene amplicons for analyses. After sampling caddisfly microbiomes, four 1L polypropylene bottles of stream water were collected in close proximity (~1-2 m) from the site of cobble collection, placed in a small cooler, and filtered within one hour through 0.2 μm pore size Sterivex™ filter cartridges using a peristaltic pump. Cartridges were filled with RNA/DNA stabilizing buffer, capped, and stored frozen until DNA extraction. Only ~500 mL of stream water could be passed through each filter prior to significant decreases in pumping rate being observed. Samples of surficial, small grain (>0.1-1.0 mm) sediment were collected using a spatula and plastic weigh boat, with ~1 g of sediment scooped into a cryovial containing RNA/DNA stabilizing buffer. All sample-containing cryovials and filter cartridges were stored on ice in a cooler during transport to the lab at Montana State University (3-4 hours maximum storage time) and then frozen at -80°C until further processing.

**DNA extraction**

DNA was extracted from April and June samples using the PowerSoil® Pro Kit (QIAGEN, Inc., Hilden Germany) following the manufacturer’s protocol, with the following customizations. 1) Nets, retreats, and rock swabs were transferred directly and in whole to the kit’s bead-beating tube. 2) Larva bodies were first homogenized using a Kimble® Pellet Pestle (DWK Life Sciences Inc., Millville, NJ) before transfer to the bead-beating tube. 3) All centrifuge steps were extended to two minutes. DNA was quantified with a Qubit™ Fluorometer (ThermoFischer, Waltham, MA). DNA was quantifiable (i.e., above detection) for 99% of samples. Average DNA concentrations per sample were 16.9 and 19.4 ng/µL for larva, 7.4 and 5.6 ng/µL for nets, 35.5 and 31.0 ng/μL, and 9.6 and 4.6 ng/μL for rock swabs for t_1_ and t_2_, respectively.

**16S rRNA gene amplicon and metagenome sequencing**

DNA extracts were aliquoted to sample-specific wells of a 96-well plate and shipped on dry ice to The Georgia Genomics and Bioinformatics Core (GGBC) at the University of Georgia for PCR amplification of the V4 hypervariable region of the 16S rRNA gene followed by Illumina MiSeq sequencing. PCR was conducted using barcoded primers 515 F GTGYCAGCMGCCGCGGTAA [[3](#_ENREF_3)] and 806R GGACTACNVGGGTWTCTAAT [[4](#_ENREF_4)] in a total volume of 25 µL that contained 5 μL KAPA HiFi™ buffer (Roche, Inc., Basel, Switzerland), 0.75 μL 10 mM dNTPs, 0.5 μL KAPA HiFi HotStart DNA polymerase, 1.5 μL each of 5 μM forward and reverse primer, and 1-2 μL template DNA. Reactions were run with the recommended Illumina thermocycler protocol of 1) 95°C for 3 minutes, 2) 25 cycles of 95°C for 30 seconds, 55°C for 30 seconds, 72°C for 30 seconds, and 3) 72°C for 5 minutes. Amplicons were pooled at equimolar concentrations and sequenced (2x250 bp) on two runs (separate runs for April and June samples) using the MiSeq™ with Reagent Kit V2 chemistry in paired-end mode (2x250 bp) (Illumina, Inc., San Diego, CA).

Metagenomic sequencing was performed at GGBC using 16 samples collected on 7 April 2021 (t1) from larvae (n=4), nets (n=4), retreats (n=4), and rock swab controls (n=4). These sample subsets were chosen randomly from among the ~25 total samples collected for each sample type at t_1_. DNA template concentrations were at least 9 ng/μL for all samples in a total volume of ~28 μL (1-2 μL was used for the amplicon analysis described above). The Nextera® XT DNA preparation kit was used to prepare and barcode the 16 metagenome libraries. Each library was analyzed for size and quality on a Bioanalyzer™ 2100 fragment analyzer (Agilent, Inc. Santa Clara, CA). Libraries were further quantified (qPCR), pooled to equimolar mixtures, and run on one lane of a NextSeq® 500 using the High Output Kit v2.5 (2x150 bp).

**16S rRNA gene amplicon analysis**

16S rRNA gene amplicon data were generated for 201 samples (96 and 105 for t_1_ and t_2_, respectively, prior to rarefaction). Forward and reverse reads were processed using DADA2 (v1.20.0) in default mode in the R statistical environment (R 4.1.0) [[5-7](#_ENREF_5)]. Briefly, read filtering and trimming were done using ‘filterAndTrim’, sample inference was done using command ‘dada’ (default parameters: truncate lengths 240 and 160, max N’s allowed set to 0, truncate Q set to 2, remove phiX set to ‘TRUE’), and paired reads were combined using ‘mergePairs’. Amplicon sequence variants (ASVs) were removed of chimeras using ‘removeBimeraDenovo’ and assigned to taxonomic groups against a non-redundant version of the SILVA rRNA gene database (nr_v138) with the ‘assignTaxonomy’ function based on the Bayesian classifier in DADA2 [[8](#_ENREF_8)]. The resulting ASV file, sample-ASV table, and taxonomy table were imported into phyloseq (v1.36.0)[[6](#_ENREF_6), [9](#_ENREF_9)] as a phyloseq object using the ‘merge’ function. The initial sample matrix contained 201 samples with 58,498 ASVs. This matrix was used preliminarily to evaluate the relative abundances of chloroplast and mitochondria sequences but then modified with the following procedures. Sequences identified as chloroplast and mitochondria (based on annotations at the level of Class and Family, respectively) were identified and removed using the ‘subset_taxa’ function in phyloseq, resulting in removal of 1934 and 1485 ASVs, respectively, or 3419). Samples with fewer than 500 sequences per sample were removed, resulting in a final sample/ASV table with 197 samples (95 and 102 samples for t_1_ and t_2_, respectively) and 55,079 ASVs. After filtering, the median sequence count per sample was 30,856 (range 528-113,697). Relative ASV counts per sample were calculated in phyloseq, and the resulting relative ASV/sample matrix was used for all downstream univariate and multivariate analyses. The 55,079 ASVs were phylogenetically analyzed using ‘rtree’ to generate general local trees with the ‘ape’ package (v5.5) of R. This tree structure was again merged into the phyloseq object and used for calculation of Bray-Curtis and UniFrac (weighted and un-weighted) distances in phyloseq. Results were similar using all metrics; we therefore presented data only for analyses using unweighted UniFrac (Main Text). Three dimensional ordinations were generated using vegan (v2.5-7) with the ‘monoMDS’ function and plotted using ‘scatterplot3d’.

We used both distance-based and machine learning methods to test if microbiome composition varied among or could be predictive of sample type. First, vegan with default settings was used to conduct analysis of similarity (ANOSIM) across the entire dataset (all sample types included) and for comparisons between rock control versus bio-structure microbiomes, with the bio-structure data containing either 1) net + retreat datasets, or 2) net or retreat datasets; results of this analysis are included as Table S1. We then applied permutational multivariate analysis of variance (PERMANOVA) using ‘adonis2’ in vegan with pairwise tests between each of the four sample types. We performed these tests using three different input matrices consisting of t_1_ only (95 samples, all types), t_2_ only (102 samples, all types), and both t_1_ and t_2_ (197 samples, larvae excluded). PERMANOVA F statistics and p-values are listed in Table S2. Finally, supervised learning through Random Forest analysis was used to test if community taxonomic data could accurately predict samples types. We used the ‘RandomForest’ (RF) package (v4.7-1.1) in the R statistical language (v4.1.3) for supervised learning using ASVs classified at the genus level (n=814) and samples (n=197) rarefied to 500 seqs/sample, performing 6 different classification tests (t_1_ only, t_2_ only, t_1_ and t_2_, t_1_ no larvae, t_2_ no larvae, t_1_ and t_2_ no larvae. Resulting out-of-bag (OOB) error rates and class errors are listed in Table S3.

We also tested for differential abundance of genera between sample types using one-way analysis of variance (ANOVA). Sample/genera matrices were produced using the ‘tax_glom’ function in phyloseq, with 814 genera observed. Differential abundance between nets or retreats versus rock control datasets was assessed using one-way ANOVA with Tukey’s Honest Significant Difference post-hoc testing in base R with the ‘aov’ and ‘TukeyHSD’ functions, respectively (p<0.05) [[10](#_ENREF_10)].

**Phylogenetic placement of *Nitrospira* ASVs**

The phylogentic relationship among nitrite-oxidizing and complete ammonia-oxidizing *Nitrospira* bacteria was analyzed in ARB (version 7) using sequences from whole genome assemblies (described below), the SILVA non-redundant database (version 138 nr), and ASVs recovered from this study [[8](#_ENREF_8), [11](#_ENREF_11)]. *Nitrospira inopinata*, *Ca.* N. nitrificans, *Ca.* N. nitrosa, and *Ca.* N. kreftii 16S rRNA genes were identified in whole genome assemblies (described below) in the Anvi’o software and then imported into ARB and automatically aligned [[12](#_ENREF_12), [13](#_ENREF_13)]. The *Nitrospira* ASV identified in the amplicon data was imported into ARB, automatically aligned with the ‘automated alignment tool’, with further manual curation. All other included 16S rRNA genes were part of the SILVA138 database, and selected for comparisons due to their close relationship to the ASV from this study. Maximum likelihood analyses were performed using the ‘PHYML-20130708’ function with bootstrap support values based on approximate Bayes branch support.

**Metagenome analysis**

*Screening for marker genes*

Shotgun metagenomes were analyzed to compare the relative abundances of microbial functional genes. Our goal was to assess the distribution of biogeochemically relevant marker genes, not to comprehensively identify all genetic features that might segregate among sample types. We therefore queried predicted open-reading frames against a database of 51 established marker genes of diverse aerobic and anaerobic metabolisms. This database was compiled and previously vetted in surveys of trace gas-consuming microorganisms [[14-16](#_ENREF_14)]. The strength of the database is its reliance on conserved domain features in each marker gene [[14](#_ENREF_14)]. A list of genes in the database is included below. The database can be accessed through FigShare [[15](#_ENREF_15)].

The analysis of the 16 metagenomes from this study proceeded as follows. Sickle was used to identify and trim low quality bases from raw forward and reverse reads using default settings. Reads were assembled using MEGAHIT (v1.2.9) with default settings [[17](#_ENREF_17)]. Prodigal (v2.6.3) was used to predict genes using the ‘meta’ (i.e., metagenomics) flag [[18](#_ENREF_18)]. The resulting ‘ffn’ files (or gene FASTA files) were used to query the 51-marker gene database using DIAMOND BLASTX [[19](#_ENREF_19)], applying a bit score cutoff of 50 and amino acid identity cutoff of 60%. We estimated genome sizes and genome equivalents using MicrobeCensus (v 1.1.0) [[20](#_ENREF_20)]. Reads per kilobase genome equivalent (RPKG) were calculated using the genome equivalent estimates from MicrobeCensus and gene counts for each of the 51 functional genes as performed previously [[21](#_ENREF_21)]. Gene frequencies for each marker were also evaluated in unassembled sequence data. For these analyses, forward fastq files (cleaned with sickle) were compared to the same database (50 markers) as for the assembled dataset. Average genome size estimates were made using MicrobeCensus. Genes were then normalized using reads per kilobase million base par (RPKM) or reads per kilobase genome equivalent (RPKG). Briefly, if a gene (referred to as *I*) of length (5000 bp or 5 kbp) is observed once in a metagenome with 5 genome equivalents its RPKG value would be:

RPKG for gene *I* = (1/5)/5 = 0.04

Tests for significant differences in marker gene frequency between sample types – focusing specifically on nets or retreats versus rock control datasets - were performed using one-way ANOVA with post-hoc analysis using Tukey’s HSD test. Boxplot frequencies of each functional gene were plotted individually for each gene in base R.

The custom database described above includes the following 51 functional genes, with gene abbreviation, number of representative sequences in the database, and average amino acid length in parentheses:

acetyl CoA synthase (*acs*B, 371, 635.5), ATP-citrate lyase (*acl*B, 68, 424.9), ammonia monooxygenase (*amo*, 66, 254.2), arsenite oxidase (*aro*, 543, 849.4), arsenate reductase (*ars*C, 20,286, 126.1), anaerobic sulfite reductase (*asr*A, 84, 341.6), F-type ATP synthase (*atp*A, 68, 424.9), cytochrome *cbb*_3_ oxidase (*cbb*3, 11,042, 497.1), anaerobic carbon monoxide dehydrogenase (*coos*, 300, 647.3), cytochrome aa_3_ oxidase (*cox*A, 22,832, 570.8), carbon monoxide dehydrogenase (*cox*L, 709, 800.7), iron oxidizing cytochrome (*cyc*2, 111, 449.2), cytochrome bo_3_ oxidase (*cyo*A, 8,201, 319.5), cytochrome bd oxidase (*cyd*A, 36,188, 483.8), dissimilatory sulfite reductase (*dsr*A, 275, 423.7), flavocytochrome c sulfide dehydrogenase (FCC, 428.7, 99), FeFe hydrogenase (FeFe *hyb*, 1,221, 203.3), Fe hydrogenase (Fe *hyd* 25, 347.1), fumarate reductase (*frd*A, 46, 602.7), crenarchaeotal 4-hydroxybutyryl-CoA synthetase (*hbs*C, 12, 471.8), Thaumarchaeota 4-hydroxybutyryl-CoA synthetase (*hbs*T, 10, 700.7), hydrazine synthase (*hsz*A, 14, 730.1), isoprene monooxygenase subunit A (*isomono*, 26, 501.5), malonyl-CoA reductase (*mcr*, 6, 1,222.8), methyl CoM reductase (*mcr*A, 195, 562.7), soluble methane monooxygenase (*mmo*, 21, 526.6), decaheme iron reductase (*mtr*B, 41, 710.3), periplasmic nitrate reductase (*nap*A, 202, 830.2), dissimilatory nitrate reductase (*nar*G, 277, 1,229), NiFe hydrogenase (NiFe *hyb*, 331, 504.7), nitrogenase (*nif*H, 1,271, 286.3), copper containing nitrite reductase (*nir*K, 338, 453.5), cytochrome cd-1 nitrite reductase (*nir*S, 164, 574), nitrous oxide reductase (*nos*Z, 403, 662.9), ammonia-forming nitrite reductase (*nrf*A, 66, 254.2), nitric oxide reductase (*nor*B, 330, 689.4), NADH-ubiquinone oxidoreductase (*nuo*F), nitrite oxidoreductase (*nxr*A, 32, 1,151.5), polyheme iron reductase (*omc*B, 18, 759.5), particulate methane monooxygenase (*pmo*A, 85, 250.4), photosystem I reaction centre (*psa*A, 54, 741.1), photosystem II reaction centre (*psb*A, 121, 337.3), ribulose-1,5-bisphosphate carboxylase/oxygenase (RubisCO, *rbc*L, 707, 479), reductive dehalogenase (*rdh*A, 46, 483.1), microbial rhodopsin (*rho*, 189, 259.7), succinate dehydrogenase (*sdh*A, 350, 613.9), sulfur oxygenase reductase (*sor*, 17, 310.6), thiosulfohydrolase (*sox*B, 235, 573.1), sulfide quinone oxidoreductase (*sqr*, 333, 422.4), selenate reductase (*ygf*K, 2,972, 1,021.3), formate dehydrogenase (*fdh*A, 962.4, 12830).

*Screening for commamox genes*

In a separate analysis, we compared screened for genes associated with complete ammonia oxidation (commamox) using a custom database containing amino acid sequences for ammonia monooxygenase (*amo*A, n=3, gene length = 281 aa), alpha (*nxr*A, 3, 1145) and beta (*nxr*B, 3, 429) nitrite oxidoreductase, hydroxylamine oxidoreductase (*hao*B, 3, 575), and ATP-citrate lyase (*acl*B, 3 606) from known commamox representatives Nitrospira inopinata [[22](#_ENREF_22), [23](#_ENREF_23)], *Ca.* N. nitrificans [[24](#_ENREF_24)], *Ca.* N. nitrosa [[24](#_ENREF_24)], and *Ca.* N. kreftii [[25](#_ENREF_25)]. Metagenomes (genes from assembled contigs or unassembled fastq reads) were compared to these proteins using DIAMOND-BLASTX as described above with a higher percent identity (90%). Hits were then normalized using average genome size estimates via MicrobeCensus. *amo*A genes identified in BLASTX comparisons of unassembled fastq files were imported into ARB using the *amo*A database specific for complete ammonia-oxidizing bacteria [[26](#_ENREF_26)]. Short fragments (149 bp) were then aligned using the automated alignment tool with manual curation. A maximum likelihood tree was then constructed and bootstrapped using approximate Bayes branch supports.

**Binning of genomes from metagenomes**

Assembled contigs were binned into metagenome-assembled genomes (MAGs) using MaxBin (v2.7) [[27](#_ENREF_27)]. MAGs were evaluated for completion and taxonomy using standard workflows, specifically, ‘anvi-estimate-scg-taxonomy’ (minimum ribosomal identity modified to 60%), ‘anvi-estimate-genome-completion’ and ‘anvi-estimate-genome-taxonomy’ in Anvi’o (v7.1) [[12](#_ENREF_12)]. This analysis generated 458 MAGs produced using the default assembly settings (1 kbp minimum contig length); statistics for these 458 MAGs can be found on GitHub (<https://github.com/TonyMane/caddis/blob/main/all_MAGs.txt>). Due to high redundancy observed among these MAGs, each MAG was filtered to remove contigs less than 2 kbp, then re-evaluated for completion/redundancy and the presence of biogeochemically relevant genes (i.e., the 50 markers and described above). This analysis identified five MAGs with less than 5% redundancy that harbored either *nar*G, *nir*K, *nor*B, *nos*Z (MAGs NET16_025, NET24_034, RETR11_010, RETR12_014; Table S6) or were phylogenetically associated with *Nitrospira* (MAG RETR11_027; Table S6). These MAGs were then used for competitive mapping (described below).

We confirmed the phylogenetic placement of the single *Nitrospira* MAG (RETR11_027) to evaluate its potential role in comammox (Figure S11). An Anvi’o database was constructed that included 35 other genomes of nitrite-oxidizing bacterial (n=4) and complete ammonia-oxidizing (n=4) isolates or enrichments, *Nitrospira* MAGs (n=25), and the iron-oxidizing bacterium *Leptospirillum ferriphilum* (Class Nitrospira) (the later was used as an outgroup) [12]. These genomes were selected based on a previously published ribosomal protein tree [[28](#_ENREF_28)]. The database was then placed through the Anvi’o-phylogenomic workflow. Briefly, Hidden Markov Model (HMMs) were then called against the default ribosomal protein database (‘Bacteria_71’, function ‘anvi-get-sequences-for-hmm-hits’. 46 shared single copy marker genes were then concatenated (function ‘anvi-get-sequences-for-hmm-hits’. These genes included Ribonuclease_P, Ribosom_S12_S23, Ribosomal_L1, Ribosomal_L13, Ribosomal_L14, Ribosomal_L16, Ribosomal_L17, Ribosomal_L18p, Ribosomal_L19, Ribosomal_L2,Ribosomal_L20, Ribosomal_L21p, Ribosomal_L22, Ribosomal_L23, Ribosomal_L27,Ribosomal_L27A, Ribosomal_L28, Ribosomal_L29, Ribosomal_L3, Ribosomal_L32p,Ribosomal_L35p, Ribosomal_L4, Ribosomal_L5, Ribosomal_L6, Ribosomal_L9_C, Ribosomal_S10, Ribosomal_S11, Ribosomal_S13, Ribosomal_S15, Ribosomal_S16, Ribosomal_S17, Ribosomal_S19, Ribosomal_S2, Ribosomal_S20p, Ribosomal_S3_C, Ribosomal_S6, Ribosomal_S7, Ribosomal_S8, Ribosomal_S9, RsfS, RuvX, SecE, SecG, SecY, SmpB, TsaE, UPF0054, YajC, eIF-1a, ribosomal_L24, tRNA-synt_1d,

tRNA_m1G_MT). Using this concatenated protein FASTA file, a phylogeny was reconstructed using ‘phylogeny.fr’ with default settings. Briefly, this involved aligned with MUSCLE, curation with Gblocks, and subsequent tree calculation with PhyML [[29](#_ENREF_29)], with bootstrapping based on the approximate likelihood ratio [[30](#_ENREF_30)].

**Read mapping, truncated average depth filtering, and percent calculations**

We mapped metagenome reads to the *Nitrospira* MAG and to four MAGs harboring denitrification-associated genes *nar*G (n=1), *nor*B (n=1), *nir*K (n=1), *nos*Z (n=2) (total of n=5 MAGs, see Table S6). The workflows used for competitive read mapping are identical to those described previously and available at the following GitHub repository [[31](#_ENREF_31)]: https://github.com/rotheconrad/00_in-situ_GeneCoverage/.

First, FASTA file headers were modified to denote each genome by its MAG name. Then all 5 genomes were concatenated into a single database. The database was made searchable (‘makeblastdb’ in the MagicBlast version 1.7) [[32](#_ENREF_32)]. Post-cleaned forward fastq files from each of the 16 metagenomes were then queried against this database using MagicBlast with default settings. BLAST results were then filtered using ‘01c_MagicBlast_ShortRead_Filter.py’ and the default settings (percent match 0.9, read length 70 bp). The filtered BLAST results were then de-concatenated to retrieve each MAG. Average nucleotide identity and coverage were calculated using ‘03a_MagicBlast_CoverageMagic.py’ and the default settings (percent ID alignment 95%, truncate value 80). ‘05_MagicBlast_CoverageMagic_CombineGenomeStats.py’ was then used to create tab-delimited flat (tsv) files of genome coverage and truncated average depth (TAD) values per genome. The percent abundance of each MAG in a metagenome was calculated as:

MAG % = {(TAD value * genome size in bp)/(metagenome size in bp)}*100

**Data availability**

All sequence data generated in this study are available through the NCBI Sequence Read Archive under BioProject PRJNA834817.

**SUPPLEMENTARY DISCUSSION**

**Sediment and stream water microbiomes**

Microbiomes associated with streambed surficial sediment (fine grains less than 0.1 mm) and channel water from t_1_ and t_2_ were taxonomically distinct from those of caddisfly bio-structures and rock controls, clearly separating from other sample types in ordination space based on unweighted UniFrac distances (Figure S5). While the low number of sediment and water samples made statistical analyses difficult, the overall trends in beta diversity suggest that caddisfly-associated microbiomes are distinct from those of the surrounding stream environment.

**Dissimilatory sulfite reductase**

Dissimilatory sulfite reductase (*dsr*) is a reversible enzyme used to catalyze sulfite reduction to hydrogen sulfide in sulfate-reducing bacteria and archaea. The reverse reaction is used by diverse sulfide-oxidizing bacteria. Reductive versus oxidative *dsr* are phylogenetically distinguishable [[33](#_ENREF_33)]. The *dsr* sequences (n = 275, see above) included in the database used in our metagenomic analysis are primarily from physiologically characterized sulfate or sulfide-oxidizing taxa, but also include environmental sequences from metagenome-assembled genomes (MAGs). These *dsr* sequences were phylogenetically evaluated previously and flagged as oxidative or reductive [[15](#_ENREF_15)]. Interestingly, oxidative *dsr* sequences were detected exclusively in rock, net, and retreat metagenomes, whereas reductive *dsr* sequences were detected exclusively in larva metagenomes. Approximately 90% of the oxidative sequences detected in nets, retreats, or rocks displayed highest % amino acid similarity to homologs from environmental metagenomes, specifically to genes from Betaproteobacterial MAGs from a freshwater aquifer near Rifle, Colorado [[2](#_ENREF_2)]. In contrast, 100% of reductive sequences detected in larvae were associated with *Desulfovibrio* species (either *D. litoralis* or *D. cuneatus*). *Desulfovibrio* is a genus of known sulfate-reducing bacteria of the phylum Thermodesulfobacteriota.

**Supplementary figure and table legends.**

Figure S1. Microbiome taxonomic composition varies based on sample type (excluding larva-associated datasets). Clustering is based on NMDS analysis of unweighted UniFrac distances based on 16S rRNA gene amplicon sequence variants recovered at t_1_ (April 7, A) and t_2_ (June 2, B).

Figure S2. Microbiome dispersion variance based on sample type (larva-associated datasets excluded). Dispersion is measured as distance from centroid calculated using unweighted UniFrac distances based on 16S rRNA gene amplicon sequence variants recovered at t_1_ (April 7, A) and t_2_ (June 2, B). The bold line is the sample mean; the boxed region is the interquartile range (IQR); top and bottom whiskers indicate [Q3 + 1.5*IQR] and [Q1 - 1.5*IQR], respectively; and outliers are marked by open circles.

Figure S3. Percentage abundance of reads identified as chloroplast in origin at t_1_ (April 7, A) and t_2_ (June 2, B). The bold line is the sample mean; the boxed region is the interquartile range (IQR); top and bottom whiskers indicate [Q3 + 1.5*IQR] and [Q1 - 1.5*IQR], respectively; and outliers are marked by open circles.

Figure S4. Microbiome taxonomic composition varies based on sample type, including water and sediment samples. Clustering is based on NMDS analysis of unweighted UniFrac distances based on 16S rRNA gene amplicon sequence variants recovered at t_1_ (April 7, A) and t_2_ (June 2, B). Panels C and D show the same NMDS ordinations, but rotated to be shown in two dimensions.

Figure S5. Microbial taxa of biogeochemical relevance vary in abundance among sample types. Abundance is expressed as a percentage of total 16S rRNA gene amplicons at t_1_ (April 7, A-E) and t_2_ (June 2, F-J). None of the included 10 taxonomic groups exceeded 1% of total reads. At t_1_, four of the five taxa were significantly enriched (one way ANOVA with Tukey’s HSD post-hoc testing) in either retreats or nets compared to rocks; at t_2_, abundance differences were not significant for any taxon. *Methanosarcina* (A, F), *Methanoregula* (B, G), *Methanobacterium* (C, H), *Candidatus* Nitrosopumilus (D, I), and ammonia/nitrite oxidizing *Nitrospira* (E, J). Asterisks (*) indicate significant enrichment in nets or retreats versus rocks (one way ANOVA with TukeyHSD post-hoc testing).

Figure S6. Denitrification-associated genes vary in abundance among sample types. Abundance is expressed as reads per kilobase genome equivalent (RPKG). Genes involved in denitrification were identified through DIAMOND-BLASTX comparisons of unassembled forward fastq files directly against the 51 gene database (see Methods). Panels A-E show genes encoding enzymes of the denitrification pathway: nitrate reductase (*narG*) (A), cytochrome cd-1 nitrite reductase (*nirS*) (B), copper containing nitrite reductase (*nirK*) (C), nitric oxide reductase (D), nitrous oxide reductase (*nosZ*) (E). The plots show results for n = 4 metagenomes per sample type. The bold line is the sample mean; the boxed region is the interquartile range (IQR); top and bottom whiskers indicate [Q3 + 1.5*IQR] and [Q1 - 1.5*IQR], respectively; and outliers are marked by open circles.

Figure S7. Denitrification gene-carrying MAGs vary in abundance among sample types Abundance is expressed as a percentage of the total reads recruited to each MAG, as described in the Methods. MAGs NET24_34 (A, *nirK* containing), RETR11_010 (B, *nosZ*), RETR12_14 (C, *nos*Z), NET16_025 (D, *narG*), RETR11_27 (E, *nxrA*). The plots show results for n = 4 metagenomes per sample type. The bold line is the sample mean; the boxed region is the interquartile range (IQR); top and bottom whiskers indicate [Q3 + 1.5*IQR] and [Q1 - 1.5*IQR], respectively; and outliers are marked by open circles.

Figure S8. Non-denitrifier/atypical (A) and denitrifier/typical (B) nitrous oxide reductase genes (*nos*Z) are enriched in net and retreat microbiomes compared to rock control and larval caddisfly microbiomes. Genes were identified from assembled contigs and used in comparisons to the *nos*Z database. Vertical axes are in reads per kilobase genome equivalent (RPKG), although the scales differ between A and B. The plots show results for n = 4 metagenomes per sample type. The bold line is the sample mean; the boxed region is the interquartile range (IQR); top and bottom whiskers indicate [Q3 + 1.5*IQR] and [Q1 - 1.5*IQR], respectively; and outliers are marked by open circles.

Figure S9. *Nitrospira* 16S rRNA (A) and *amoA* (B) genes (recovered in amplicon and metagenomic data, respectively) cluster with those of complete ammonia-oxidizing bacteria (comommox, purple) and nitrite-oxidizing bacteria (green). 16S rRNA and *amoA* genes from this study are indicated in red. Genbank accession numbers, molecule types, informal clone names, and environmental origins are listed. Both phylogenetic trees were constructed using maximum likelihood with bootstrap support values based on approximate Bayes analyses.

Figure S10. Genes associated with complete ammonia oxidation are enriched in retreat microbiomes in both unassembled (A-E) and assembled datasets (H-J). Ammonia monooxygenase (*amoA*, A,E), alpha subunit nitrite oxidoreductase (*nxrA*, B, G), beta subunit nitrite oxidoreductase (*nxrB*, C, H), hydroxylamine oxidoreductase (*haoB*, D, I), and alpha subunit ATP-citrate lyase (*aclA*, E, J). Abundance is expressed as reads per kilobase genome equivalent (RPKG). The plots show results for n = 4 metagenomes per sample type. The bold line is the sample mean; the boxed region is the interquartile range (IQR); top and bottom whiskers indicate [Q3 + 1.5*IQR] and [Q1 - 1.5*IQR], respectively; and outliers are marked by open circles.

Figure S11. A retreat-associated MAG (arrow) was phylogenetically associated with *Nitrospira* Clade B (SI Table 6) and closely related to MAGs from freshwater sand filters. The phylogeny was constructed using maximum likelihood analysis of concatenated ribosomal marker genes (n=39 shared markers) with bootstrap values based on the approximate likelihood ratio test (aLRT). Nitrite oxidizing (blue) and complete ammonia-oxidizing (red) bacteria are denoted. MAGs (black) are listed. MAG-RETR11_27 (bold, black) from this study is denoted as such. MAG identifiers and their environmental origins are listed.

Table S1. Results of analysis of similarity (ANOSIM) comparing 16S rRNA gene composition among microbiome habitats. ‘Groups Tested’ lists the microhabitats (larvae, nets, retreats, or rocks) included in each test. ‘Samples per group’ describes the number of 16S rRNA gene amplicon datasets included per sample group in a given test. ANOSIM R values for weighted UniFrac (‘w-UniFrac’), un-weighted UniFrac (‘UniFrac’), and Bray-Curtis distance matrices are reported. ‘Sampling date’ indicates whether the datasets represent t_1_, t_2_, t_1_ + t_2,_ or t_1_ versus t_2_ collection dates.

Table S2. Results of permutational multivariate analysis of variance (PERMANOVA) comparing 16S rRNA gene composition among microbiome habitats. Degrees of Freedom (DF), Sum of Squares (SumofSqs), R^2^, pseudo F statistic (F), p-value for F statistic (Pr(>F)).

Table S3. Results of Random Forest analysis of 16S rRNA gene composition among microbiome habitats. Out of Bag (OOB) error rates and confusion matrices are listed in A-F. ‘All sample types’ includes larvae, nets, retreats, and rocks. ‘No larvae’ means that larva microbiomes were excluded. The tests involve datasets representing t_1_, t_2_, or t_1_ + t_2_. Class (or classification) errors are listed in the far right column for each test.

Table S4 (TOO LARGE FOR PDF, SEE EXCEL SHEET). Mean Decrease in Gini coefficient (MDG) results for 814 genera used in Random Forest analysis. The test evaluated all 197 samples for their affiliation in a given sample group (larvae, rocks, nets, retreats). MDG scores are sorted in decreasing order. ‘Genus ASV Rep’ (column 1) lists the ASV number used in representing a given genus. ‘Mean Decrease Gini’ (column 2) is the Mean Decrease in Gini coefficient. ‘Genus’ (column 3) lists the genus name of the Genus ASV Rep (column 3). Red taxa were identified ANOVA statistical analysis as being significantly enriched in a caddisfly biostructure.

Table S5. Metagenome sequencing information. ‘Sample Name’ indicates the sample type (larva, rock, net, retreat) and internal numbering used in the study. Sequencing depth reports the raw depth in millions of base-pairs sequenced (Mbp) per sample. Maximum Contig Length (Max Contig Length) reports the length of the largest contig after assembly with MEGAHIT. Average contig length is the mean contig length after assembly with MEGAHIT. N50 describes the weighted average such that 50% of the assembled contigs are equal to or greater than this length.

Table S6 (TOO LARGE FOR PDF, SEE EXCEL SHEET). Statistics for NOx-reducing (A) and nitrifying (B) genomes. Columns A-P are directly exported from Anvi’o using ‘estimate-genome-completeness’ and ‘estimate-genome-taxonomy’ functions, respectively. Extended columns Q-W (A) and Q-V (B) are genes involved in reductive NOx (A) nitrification (B) identified through BLASTX comparisons.

**Supplementary figures and tables**

Figure S1.

Figure S2.

Figure S3.

Figure S4.

Figure S5.

Figure S6.

Figure S7.

Figure S8.

Figure S9.

Figure S10.

Figure S11.


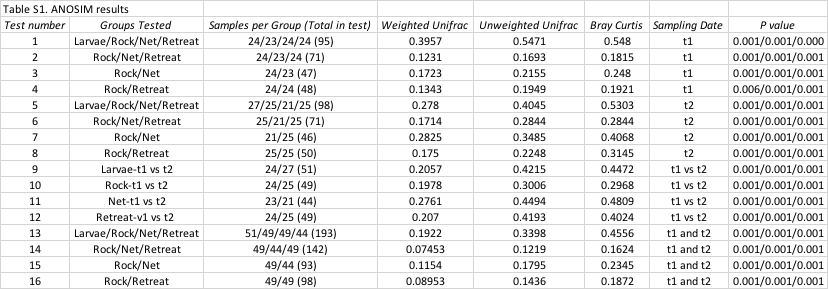


Table S1.


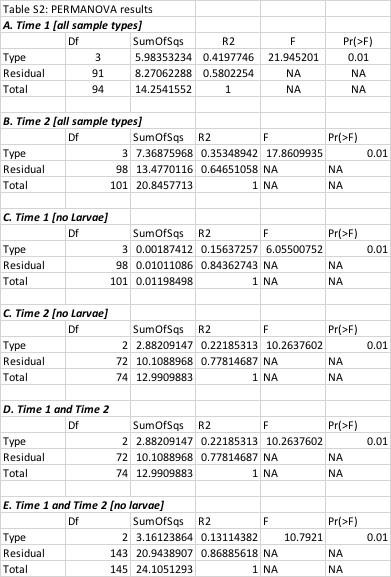


Table S2.


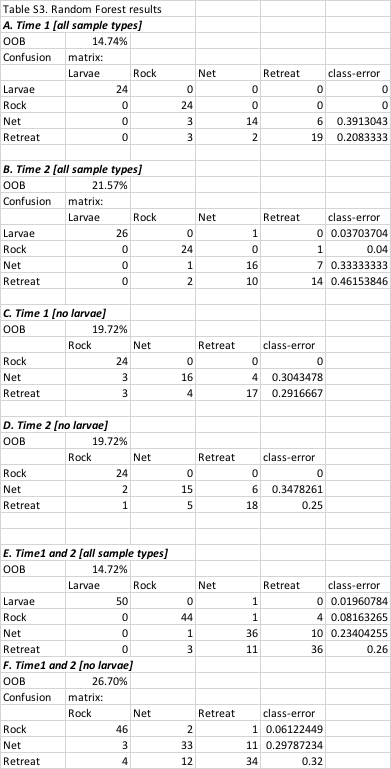


Table S3.

Table S4. See Excel file.


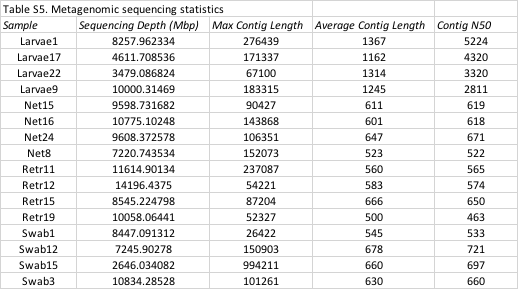


Table S5.

Table S6. See Excel file.

1. Tumolo, B.B., L.K. Albertson, W.F. Cross, M.D. Daniels, and L.S. Sklar, *Occupied and abandoned structures from ecosystem engineering differentially facilitate stream community colonization.* Ecosphere, 2019. **10**(5): p. e02734.

2. Hug, L.A., B.C. Thomas, C.T. Brown, K.R. Frischkorn, K.H. Williams, S.G. Tringe et al., *Aquifer environment selects for microbial species cohorts in sediment and groundwater.* The ISME journal, 2015. **9**(8): p. 1846-1856.

3. Apprill, A., S. McNally, R. Parsons, L. Weber, *Minor revision to V4 region SSU rRNA 806R gene primer greatly increases detection of SAR11 bacterioplankton.* Aquatic Microbial Ecology, 2015. **75**(2): p. 129-137.

4. Parada, A.E., D.M. Needham, and J.A. Fuhrman, *Every base matters: assessing small subunit rRNA primers for marine microbiomes with mock communities, time series and global field samples.* Environmental Microbiology, 2016. **18**(5): p. 1403-1414.

5. Callahan, B.J., P.J. McMurdie, M.J. Rosen, A.W. Han, A.J.A. Johnson, S.P. Holmes, *DADA2: High-resolution sample inference from Illumina amplicon data.* Nature Methods, 2016. **13**(7): p. 581-583.

6. McMurdie, P.J. and S. Holmes, *Shiny-phyloseq: Web application for interactive microbiome analysis with provenance tracking.* Bioinformatics, 2014. **31**(2): p. 282-283.

7. (2022), R.C.T., *R: A language and environment for statistical computing, Vienna, Austria.* 2022.

8. Quast, C., E. Pruesse, P. Yilmaz, J. Gerken, T. Schweer, P. Yarza, et al., *The SILVA ribosomal RNA gene database project: improved data processing and web-based tools.* Nucleic acids research, 2013. **41**(Database issue): p. D590-D596.

9. McMurdie, P.J. and S. Holmes, *phyloseq: An R Package for Reproducible Interactive Analysis and Graphics of Microbiome Census Data.* PLOS ONE, 2013. **8**(4): p. e61217.

10. Tukey, J.W., *The Philosophy of Multiple Comparisons.* Statistical Science, 1991. **6**(1): p. 100-116, 17.

11. Ludwig, W., O. Strunk, R. Westram, L. Richeter, H. Meier, Y.A. Buchner et al., *ARB: a software environment for sequence data.* Nucleic acids research, 2004. **32**(4): p. 1363-1371.

12. Eren, A.M., E. Kiefl, A. Shaiber, I. Veseli, S.E. Miller, M.S. Schechter et al., *Community-led, integrated, reproducible multi-omics with anvi’o.* Nature Microbiology, 2021. **6**(1): p. 3-6.

13. Eren, A.M., Ö.C. Esen, C. Quince, J.H. Vineis, H.G. Morrison, M.L. Sogin et al., *Anvi'o: an advanced analysis and visualization platformfor 'omics data.* Peerj, 2015. **3**.

14. Søndergaard, D., C.N.S. Pedersen, and C. Greening, *HydDB: A web tool for hydrogenase classification and analysis.* Scientific Reports, 2016. **6**(1): p. 34212.

15. Chiri, E., P.A. Nauer, R. Lappan, T. Jirapanjawat, D.W. White, K.M. Handley et al., *Termite gas emissions select for hydrogenotrophic microbial communities in termite mounds.* Proceedings of the National Academy of Sciences, 2021. **118**(30): p. e2102625118.

16. Bay, S.K., X. Dong, J.A. Bradely, P.M. Leung, R. Grinter, T. Jirapanjawat et al., *Trace gas oxidizers are widespread and active members of soil microbial communities.* Nature Microbiology, 2021. **6**(2): p. 246-256.

17. Li, D., C. Liu, R. Luo, K. Sadakane, T. Lam, *MEGAHIT: an ultra-fast single-node solution for large and complex metagenomics assembly via succinct de Bruijn graph.* Bioinformatics, 2015. **31**(10): p. 1674-1676.

18. Hyatt, D., G. Chen, P.F. LoCascio, M.L. Land, F.W. Larimer, L.J. Hauser, *Prodigal: prokaryotic gene recognition and translation initiation site identification.* BMC Bioinformatics, 2010. **11**(1): p. 119.

19. Buchfink, B., K. Reuter, and H.-G. Drost, *Sensitive protein alignments at tree-of-life scale using DIAMOND.* Nature Methods, 2021. **18**(4): p. 366-368.

20. Nayfach, S. and K.S. Pollard, *Average genome size estimation improves comparative metagenomics and sheds light on the functional ecology of the human microbiome.* Genome Biology, 2015. **16**(1): p. 51.

21. Bertagnolli, A.D., K.T. Konstantinidis, and F.J. Stewart, *Non-denitrifier nitrous oxide reductases dominate marine biomes.* Environ Microbiol Rep, 2020. **12**(6): p. 681-692.

22. Daims, H., et al., *Complete nitrification by Nitrospira bacteria.* Nature, 2015. **528**(7583): p. 504-509.

23. Kits, K.D., C.J. Sedlacek, E.V. Lebedeva, P. Han, A. Bulaev, P. Pjevac et al., *Kinetic analysis of a complete nitrifier reveals an oligotrophic lifestyle.* Nature, 2017. **549**(7671): p. 269-272.

24. van Kessel, M.A.H.J., D.R. Speth, M. Albertsen, P.H. Nielsen, H. J. M. Op den Camp, B. Kartal et al., *Complete nitrification by a single microorganism.* Nature, 2015. **528**(7583): p. 555-559.

25. Sakoula, D., H. Koch, J. Frank, M.S.M. Jetten, M.A.H.J. van Kessel et al., *Enrichment and physiological characterization of a novel comammox Nitrospira indicates ammonium inhibition of complete nitrification.* The ISME Journal, 2021. **15**(4): p. 1010-1024.

26. Pjevac, P., C. Schauberger, L. Poghosyan, C.W. Herbold, M.A.H.J. van Kessel, A. Daebler et al., *AmoA-Targeted Polymerase Chain Reaction Primers for the Specific Detection and Quantification of Comammox Nitrospira in the Environment.* Frontiers in Microbiology, 2017. **8**.

27. Wu, Y.-W., B.A. Simmons, and S.W. Singer, *MaxBin 2.0: an automated binning algorithm to recover genomes from multiple metagenomic datasets.* Bioinformatics, 2015. **32**(4): p. 605-607.

28. Palomo, A., Dechesne, O.X. Cordero, B.F. Smets, *Evolutionary Ecology of Natural Comammox Nitrospira Populations.* mSystems, 2022. **7**(1): p. e01139-21.

29. Dereeper, A., V. Guignon, G. Blanc, S. Audic, S. Buffet, F. Chevenet et al., *Phylogeny.fr: robust phylogenetic analysis for the non-specialist.* Nucleic Acids Res, 2008. **36**(Web Server issue): p. W465-9.

30. Anisimova, M. and O. Gascuel, *Approximate likelihood-ratio test for branches: A fast, accurate, and powerful alternative.* Syst Biol, 2006. **55**(4): p. 539-52.

31. Conrad, R.E., T. Viver, J.F. Gago, J.K. Hatt, S.N. Venter, R. Rossolo-Mora et al., *Toward quantifying the adaptive role of bacterial pangenomes during environmental perturbations.* The ISME Journal, 2022. **16**(5): p. 1222-1234.

32. Boratyn, G.M., J. Thierry-Mieg, D. Thierry-Mieg, B. Busby, and T.L. Madden, *Magic-BLAST, an accurate RNA-seq aligner for long and short reads.* BMC Bioinformatics, 2019. **20**(1): p. 405.

33. Müller, A.L., K.U. Kjeldsen, T. Rattei, M. Pester, and A. Loy, *Phylogenetic and environmental diversity of DsrAB-type dissimilatory (bi)sulfite reductases.* The ISME Journal, 2015. **9**(5): p. 1152-1165.
